# Vestibulo-Oculomotor Reflex Dysfunction in Children with Cerebral Palsy Correlates with Gross Motor Function Classification System

**DOI:** 10.3390/audiolres15020021

**Published:** 2025-02-25

**Authors:** Laura Casagrande Conti, Nicola Ferri, Leonardo Manzari, Tommaso Lelli, Maria Mangeruga, Margherita Dal Piaz, Andrea Manzotti, Luca Verrecchia, Marco Tramontano

**Affiliations:** 1 Santa Lucia Foundation, Scientific Institute for Research and Health Care, 00179 Rome, Italy; laura.casagrandeconti@gmail.com (L.C.C.); tommi.lelli99@gmail.com (T.L.); m.mangeruga@hsantalucia.it (M.M.); m.dalpiaz@hsantalucia.it (M.D.P.); 2 Department of Biomedical and Neuromotor Sciences, University of Bologna, 40138 Bologna, Italy; marco.tramontano@unibo.it; 3 MSA ENT Academy Center, 03043 Cassino, Italy; lmanzari1962@gmail.com; 4 Research Department, SOMA, Istituto Osteopatia Milano, 20126 Milan, Italy; manzotti.andrea68@gmail.com; 5 RAISE Lab, Clinical-Based Human Research Department, Foundation COME Collaboration, 65121 Pescara, Italy; 6 Karolinska Ear and Hearing, Karolinska University Hospital, 17176 Stockholm, Sweden; luca.verrecchia@regionstockholm.se; 7 Unit of Occupational Medicine, IRCCS Azienda Ospedaliero-Universitaria di Bologna, 40138 Bologna, Italy

**Keywords:** cerebral palsy, head impulse test, postural balance, rehabilitation, vestibular system

## Abstract

Background/Objectives: This study aims to evaluate the feasibility of the angular vestibulo-ocular reflex (aVOR) function assessment in children with cerebral palsy (CP) using the video Head Impulse Test (vHIT) and to investigate how vestibular impairments correlate with functional motor ability. Methods: This cross-sectional study included children diagnosed with CP who attempted a vestibular function assessment with the vHIT. Descriptive statistics and a subgroup analysis based on clinical expression were performed. Finally, the correlation between aVOR gain and functional motor ability was investigated. Results: Thirteen children underwent assessments of the horizontal semicircular canals. Six out of thirteen children presented at least one dysfunctional canal; overall, eight out of twenty-six horizontal semicircular canals were dysfunctional in the HIMP paradigm. A subgroup analysis revealed a dysfunctional aVOR gain in all the children presenting ataxia. The correlation analysis demonstrated a strong negative association between aVOR gain and the Gross Motor Function Classification System (−0.73 and −0.68 for the left and right horizontal canal, respectively; *p* < 0.05). Conclusions: Vestibular dysfunctions are prevalent in children with CP and strongly correlate with motor function. An instrumental vestibular assessment in children with cognitive-motor disability seems feasible, in particular for horizontal canals and the HIMP paradigm. This could be important for better framing a child’s function and enhancing the management of balance and motor impairments with more specific strategies for children with CP.

## 1. Introduction

Cerebral palsy (CP) represents one of the most common causes of physical disability in childhood, with an estimated prevalence of 2 to 3 per 1000 live births globally [1]. CP is a non-progressive disorder arising from damage to the developing fetal or infant brain and is characterized by disturbances in movement, posture, and motor function [2]. While the primary motor impairments of CP, such as spasticity, dystonia, and ataxia, are well-recognized, the role of sensory dysfunctions, particularly vestibular impairments, remains underexplored. Increasing evidence suggests that the vestibular system significantly influences motor performance, balance, and overall quality of life in children with CP [3,4]. The vestibular system, which consists of peripheral receptors in the inner ear and central processing pathways, is essential for maintaining gaze stability, spatial orientation, and postural equilibrium [5]. It provides critical sensory input that allows individuals to navigate dynamic environments effectively [5]. Dysfunction within the vestibular system can disrupt these processes, leading to impairments in gaze stabilization during rapid head movements, reduced balance control, and an increased risk of falls [6,7,8]. For children with CP, who already face significant challenges in motor coordination, the additional burden of vestibular dysfunction can severely impact their ability to engage in daily activities and participate in social and physical environments [4].

Despite its importance, vestibular dysfunction in children with CP remains poorly characterized. Research has largely focused on motor impairments and associated visual deficits, such as reduced visual acuity and strabismus, with limited attention to vestibular function [9,10,11,12,13]. However, recent advancements in diagnostic tools, particularly the video Head Impulse Test (vHIT), have made it possible to quantitatively assess the angular vestibulo-ocular reflex (aVOR) in pediatric populations [14]. The vHIT is a non-invasive tool that evaluates the functionality of the semicircular canals by measuring the gain of the aVOR during rapid head movements [15,16].

In children with CP, the potential implications of vestibular dysfunction extend beyond balance and motor coordination. Impairments in the vestibular system can affect spatial orientation, gaze stabilization during dynamic activities, and the integration of multisensory inputs necessary for efficient motor planning and execution [17]. For instance, activities such as walking, running, and playing sports require precise coordination between visual, proprioceptive, and vestibular inputs [4]. Disruption in any of these systems can compromise the child’s ability to perform these activities safely and effectively, further limiting their participation and independence [2,17].

Previous studies have demonstrated the utility of vHIT in assessing vestibular function in adults with neurological disorders [18,19,20], but its application in younger children with cognitive and motor impairments has been limited [14].

To the best of our knowledge, no studies have been conducted using vHIT in children with neurological disorders, specifically cerebral palsy. We hypothesize that although the vestibular system is developed in children with perinatal brain injury, the VOR pathways from the semicircular canals may be impaired.

This study aims to investigate the prevalence of aVOR dysfunction in children with CP and explore its relationship with functional motor abilities. Additionally, this study assesses the feasibility of using vHIT in a neurorehabilitation setting for children with CP.

## 2. Materials and Methods

### 2.1. Study Design and Setting

This cross-sectional study was approved by the Local Independent Ethics Committee (Prot. CE/2022_011) and was conducted at the pediatric neurorehabilitation center from September 2023 to September 2024. It adhered to the Strengthening the Reporting of Observational Studies in Epidemiology (STROBE) guidelines for cross-sectional studies [21].

### 2.2. Study Participants

Eligibility criteria included children between the ages of 3 and 11 years diagnosed with CP, with spastic paresis (either unilateral or bilateral) or ataxia, and a Level of Sitting Scale (LSS) [22] score greater than 4. Children with CP who could understand the task “to fix a picture on the wall” were recruited after obtaining the parents’ written informed consent. We excluded children with severe visual fixation disorders, a Southern California College of Optometry System (SCCOS) score >1, severe spasticity, and/or bone deformities.

### 2.3. Measurements

#### 2.3.1. Gross Motor Function Classification System

The functional capabilities of individuals with CP were classified according to the GMFCS [23,24]. This system categorizes individuals into five levels of mobility, ranging from level one (I), which represents the least impaired, to level five (V), which represents the most impaired. Across this spectrum of motor impairments, visual and oculomotor limitations can further restrict participation in everyday life.

#### 2.3.2. Instrumental Assessment of the VOR

The vHIT (ICS Impulse, Otometrics/Natus, Taastrup, Denmark) was used to evaluate the aVOR gain for movements in the direction and plane of the stimulation of the six semicircular canals, and the outcomes were collected for both the HIMP and SHIMP paradigms [25,26,27,28]. To ensure precision and consistency, a standardized assessment protocol was followed to minimize variability and enhance data accuracy [29]. All tests were conducted by two physiotherapists, who had undergone specialized training under the guidance of an expert clinician (LM) and were supervised throughout the study by a senior physiotherapist (MT) with extensive expertise in the vestibular system. Testing protocols included:

HIMP Paradigm: Children were instructed to fixate on a static visual target while their head was rapidly rotated by the examiner. To ensure the attention of the children, small toys and other games were used as targets.

SHIMP Paradigm: Children tracked a moving target generated by a laser attached to their head, allowing for the identification of compensatory saccades. Under normal conditions, a saccade in the opposite direction of the aVOR reflex is expected after a brief delay.

Room lighting was controlled to ensure that pupil size remained small and that there were no reflective artifacts in the pupil image throughout the head movements. To ensure the attention of the children, small toys and other games were used as targets. During each session, approximately 12 rapid, brief horizontal head impulses were administered to each side, starting from a neutral head position. The direction and timing of the head turns were unpredictable, with minimal rebound or overshot at the end of each movement, following a precise “turn and stop” technique. The head rotation amplitude was roughly 10–15°, with peak head velocities of 140–220°/s and angular accelerations between 3000–5000°/s^2^. Both head and eye velocities were recorded for each head turn. In the HIMP paradigm, the effectiveness of the VOR is typically evaluated using the gain, calculated as the ratio of eye velocity to head velocity at peak head acceleration. Under normal physiological conditions, compensatory saccades are not present.

To assess the vertical semicircular canals, the child’s head was positioned at approximately 35 degrees to the left for the RALP (Right Anterior-Left Posterior) test and 35° to the right for the LARP (Left Anterior-Right Posterior) test. This alignment ensured that the vertical canals aligned with the plane of head rotation, optimizing stimulation through head pitch movements. The clinician stabilized the patient’s head by placing one hand on top and the other under the chin. The head was moved rapidly and unpredictably within a small angle (around 10–20°), ensuring the movement involved the skull rather than the skin, which prevented the goggles from slipping and creating false eye movement artifacts.

In terms of gain ranges, for the HIMP paradigm, we considered functional aVOR gain to be between 0.76 and 1.29 for the vertical canals and between 0.80 and 1.29 for the horizontal canals [30]. In the SHIMP paradigm, we considered a functional gain range between 0.66 and 1.29 [27].

#### 2.3.3. Statistical Analysis

STATA 18 software (StataCorp, 2023, College Station, TX, USA) [31] was used for all statistical analyses. Continuous data are presented as means and standard deviations or medians and interquartile ranges, whilst categorical data are reported with frequencies. We investigated the normal distribution of data using the Shapiro-Wilk test and performed correlation analyses between aVOR gain and GMFCS level by calculating Spearman’s rank correlation coefficient. Given that GMFCS is ordinal, this non-parametric method provides a more reliable interpretation of the relationship between the two variables while accounting for potential non-linearity.

## 3. Results

A total of 20 children were recruited according to inclusion criteria. Four children were unable to follow the physiotherapist’s instructions during the test, leaving sixteen to undergo the evaluation. Despite several attempts with one child, the test could not be performed due to the goggles being an improper fit. Additionally, two children opted not to continue the assessment due to discomfort. As a result, thirteen children underwent assessments of the horizontal semicircular canals, but only one completed the evaluation on the vertical plane. Three children were also assessed using the SHIMP paradigm, while for the remaining ten children, the procedure was too difficult to perform. Demographic and clinical characteristics are reported in Table 1.

**Figure 1:**
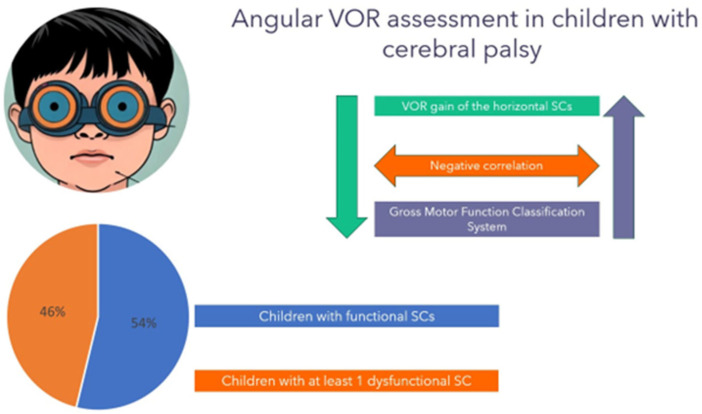
Prevalence of children with dysfunctional aVOR and correlation with GMFCS.

Six out of thirteen children who completed the horizontal canal evaluation exhibited at least one dysfunctional canal (Figure 1).

Eight out of twenty-six horizontal semicircular canals evaluated using the HIMP paradigm were dysfunctional (Table 2).

However, considering the overall sample mean, the results demonstrate a functional gain (Table 3). When analyzing the mean aVOR gain by clinical subgroups, children with ataxia presented a dysfunctional aVOR mean gain on both the left horizontal canal (gain = 0.72, 95% CI 0.13–1.31, n = 3) and the right horizontal canal (gain = 0.76, 95% CI 0.15–1.37, n = 3). In particular, half of the horizontal canals assessed in this population were dysfunctional according to normative ranges.

Three children completed the evaluation with the SHIMP paradigm, and one child showed an abnormal left horizontal canal function (gain = 0.52).

Subsequently, the correlation analysis revealed a strong negative association between aVOR gain and GMFCS (Figure 1 and Table 4), showing a decline in aVOR gain as functional impairment increases. Table 5 reports the left horizontal and right horizontal aVOR gains grouped by GMFCS levels.

## 4. Discussion

This study aimed to evaluate the presence of aVOR dysfunction in children with CP. Our findings indicate that children with CP may present a vestibular dysfunction in the horizontal semicircular canals, particularly among those with higher levels of motor impairment. The impaired aVOR gain observed in our sample highlights the potential role of vestibular assessment in a rehabilitative setting. This aligns with previous research indicating that vestibular dysfunction is prevalent in children with CP and may contribute significantly to their balance, coordination, and postural control issues [32].

Several studies have investigated the connection between vestibular function and motor abilities in children with CP, further supporting the significance of our findings. The identified correlation between aVOR gain and GMFCS levels offers valuable insights into the multisensory deficits experienced by children with CP. As motor impairment worsens, the vestibular system’s capacity to compensate for rapid head movements declines, resulting in difficulties with activities that require dynamic stability. For example, Janky et al. examined postural control and balance in children with CP, reporting that impaired vestibular function was a key factor contributing to their instability [17]. Likewise, Almutairi and colleagues [33] found that children with CP frequently exhibit deficits in sensory integration, including vestibular processing, which can substantially impact their balance and gait. These findings [17,33] align with our results, underscoring the necessity of evaluating vestibular function in this population to fully comprehend their motor impairments. Moreover, analyzing vestibular function by subgroups based on clinical expression, ataxia is the only pattern in which the mean aVOR gain is abnormal for both the right and left horizontal canals. Indeed, in this sample, 50% of the canals resulted in impairment according to the normative cut-offs. Dysfunction of aVOR gain indicates that children with ataxia may experience significant challenges with dynamic visual stability (maintaining a steady vision when moving) and spatial orientation, leading to difficulties with motor tasks that require precise balance and coordination.

Our use of the vHIT to assess the aVOR is consistent with modern approaches to vestibular evaluation. Studies such as that of Zhou and colleagues [34] highlight the efficacy of vHIT in identifying vestibular deficits in pediatric populations. The presence of abnormal aVOR gain in six of the thirteen children evaluated in our study suggests that vestibular impairment could be present in children with CP, confirming the usefulness of the vestibular evaluation in a neurorehabilitation setting [35].

Only one child completed the vertical canal assessment, highlighting the difficulty of this tool in assessing children with cognitive-motor disabilities. The feasibility of using the vHIT to assess horizontal canal function in children with CP suggests that this tool could be integrated into routine clinical evaluations. However, significant challenges remain, particularly in assessing vertical canals. The lack of properly designed goggles for children and difficulties in maintaining their attention highlights the need for innovative solutions, such as child-specific equipment or alternative testing methods, including eye-tracker devices that do not require physical goggles.

From a clinical perspective, these results support the critical role that vestibular function plays in the overall motor performance of children with CP. It is important to integrate vestibular assessments, such as the vHIT, into routine clinical evaluations to better understand the multisensory deficits these children face. Studies suggest vestibular rehabilitation can improve balance and gait in pediatric populations [36,37,38,39], and there is reason to believe similar interventions could benefit children with CP. Targeted vestibular physical therapy programs may help mitigate some of the balance issues linked to vestibular dysfunction and improve participation in activities of daily living. These findings support the inclusion of vestibular evaluations and interventions in rehabilitation programs for children with CP to optimize their motor and functional outcomes. Overall, the relationship between vestibular dysfunction and motor impairments in children with CP requires further exploration. Longitudinal studies with larger sample sizes, assessing both horizontal and vertical semicircular canals and central vestibular processing, are necessary to understand better how vestibular impairments develop over time in this population. Moreover, future studies should consider using comprehensive vestibular assessments alongside other sensory and motor evaluations to develop more rehabilitation programs for children with CP based on sensory reweighting.

Our study has some limitations. The observational design implies not being able to infer a causal link between CP and abnormal aVOR gain. The level of disability did not allow the evaluation of vertical semicircular canal function and the SHIMP paradigm. Moreover, the sample size was small, potentially compromising our findings’ robustness and generalizability. Finally, we did not perform the clinical Head Impulse test (HIT) [40], which is commonly used in everyday practice as a bedside test. A comparison with the vHIT findings could have confirmed a discrepancy between clinical and video-assisted HIT, suggesting a central lesion pattern [41]. However, it must be acknowledged that the HIT may be less useful if the pattern shown in our study can be confirmed, given that the aVOR reduction is too small to be appreciable at HIT. Furthermore, future research should address the limitations identified in this study, including a complete evaluation of the vestibular function using vestibular-evoked myogenic potentials.

## 5. Conclusions

This study contributes to the expanding evidence that individuals with neurological disorders, including children with cerebral palsy, may experience vestibular dysfunctions. Our findings demonstrate that instrumental vestibular assessment is feasible in children with cognitive-motor disabilities, but only for the horizontal canals and using the HIMP paradigm. Further research is necessary to investigate the function of vertical canals and central vestibular pathways. Integrating vestibular assessments into routine clinical practice may improve the understanding and management of balance and motor impairments in children with cerebral palsy.

## Figures and Tables

**Table 1:** Characteristics of participants (n = 13).

Female (n, %)	8 (61.54)
Age (years ± SD)	7.76 ± 2.65
Clinical characteristics (n, %)	
Ataxia	3 (25.00)
Diplegia	4 (33.33)
Hemiplegia	2 (16.67)
Other	7 (43.00)
GMFCS (n, %)	
Level I	6 (46.15)
Level II	4 (30.77)
Level III	3 (23.08)
Level IV	0 (0.00)
Level V	0 (0.00)

GMFCS: Gross Motor Function Classification System.

**Table 2:** Abnormal aVOR gain in horizontal canals during HIMP assessment (n = 13).

	Abnormal aVOR Gain (n, %)
Left Horizontal	4 (30.77)
Right Horizontal	4 (30.77)

**Table 3:** Overall aVOR gain for both HIMP and SHIMP paradigms.

HIMP aVOR Gain	Total Canals (n)	Mean ± SD	95% CIs
Left Horizontal	13	0.80 ± 0.17	[0.69, 0.90]
Right Horizontal	13	0.90 ± 0.17	[0.79, 1.01]
Left Anterior	1	0.86	NA
Right Anterior	1	1.22	NA
Left Posterior	1	0.92	NA
Right Posterior	1	0.60	NA
SHIMP aVOR gain	Total canals (n)	Mean ± SD	95% CIs
Left Horizontal	3	0.73 ± 0.19	NA
Right Horizontal	3	0.85 ± 0.07	NA

NA: not applicable.

**Table 4:** Correlation between aVOR gain and GMFCS (n = 13).

	Left Horizontal	*p*-Value	Right Horizontal	*p*-Value
GMFCS	−0.73	0.0092	−0.68	0.0114

GMFCS: Gross Motor Function Classification System.

**Table 5:** aVOR gain and GMFCS levels (n = 13).

	HIMP aVOR Gain	Total Canals (n)	Mean ± SD
GMFCS 1	Left Horizontal	6	0.91 ± 0.09
	Right Horizontal	6	1.02 ± 0.09
GMFCS 2	Left Horizontal	4	0.75 ± 0.18
	Right Horizontal	4	0.86 ± 0.17
GMFCS 3	Left Horizontal	3	0.65 ± 0.18
	Right Horizontal	3	0.73 ± 0.19

GMFCS: Gross Motor Function Classification System.

## Data Availability

Data are available upon reasonable request to the corresponding author.
